# Prevalence of syphilis and risk factors among HIV-positive men who have sex with men in Guangdong province

**DOI:** 10.3389/fpubh.2022.1025221

**Published:** 2022-11-09

**Authors:** Peizhen Zhao, Ziying Yang, Ye Zhang, Jinmei Chen, Xuezhen Fu, Weiming Tang, Jiyuan Zhou

**Affiliations:** ^1^Department of Biostatistics, State Key Laboratory of Organ Failure Research, Ministry of Education, and Guangdong Provincial Key Laboratory of Tropical Disease Research, School of Public Health, Southern Medical University, Guangzhou, China; ^2^Dermatology Hospital, Southern Medical University, Guangzhou, China; ^3^Kirby Institute, New South Wales University, Sydney, NSW, Australia; ^4^International Department of Nanjing No.13 Middle School, Nanjing, China; ^5^University of North Carolina Project-China, Guangzhou, China; ^6^Guangdong-Hong Kong-Macao Joint Laboratory for Contaminants Exposure and Health, Guangzhou, China

**Keywords:** men who have sex with men, human immunodeficiency virus, syphilis, sexually transmitted diseases, risk factors

## Abstract

**Background:**

HIV-positive men who have sex with men (MSM) tend to have high syphilis incidence. Our objective is to evaluate the prevalence of syphilis and determine the risk factors of syphilis among HIV-positive MSM.

**Methods:**

A cross-sectional study with convenience sampling was performed among HIV-positive MSM in six cities of Guangdong Province from June 2020 to August 2021. Participants completed a survey including social-demographic characteristics, sexual behaviors and self-reported syphilis, chlamydia, gonorrhea, herpes, human papillomavirus statuses after HIV diagnosis. Multivariate logistic regression was used to determine the factors associated with syphilis and sexually transmitted diseases (STD).

**Results:**

Among 944 HIV-positive MSM, 141 (14.9, 95% CI: 12.7–17.2%) men had syphilis and 220 (23.3, 95% CI: 20.6–26.0%) men had STD. Multivariate analysis indicated that MSM who met male sexual partners mainly through traditional meeting places (spa or bath house, sauna, foot or body massage parlor) in the last 6 months [adjusted Odds Ratio (a*OR*) = 2.91, 95% CI: 1.09–7.79], and who were diagnosed with herps after the HIV diagnosis (a*OR* = 3.79, 95% CI: 1.16–12.39) were more likely to have syphilis. In addition, MSM who met male sexual partners mainly through traditional meeting places in the last 6 month (a*OR* = 2.55, 95% CI: 1.01–6.42), and who had more than one male sexual partner in the last 6 months (a*OR* = 1.88, 95% CI: 1.17–3.02) were more likely to have STD.

**Conclusions:**

The prevalence of syphilis and other STDs is relatively high among HIV-positive MSM in southern China. Routine syphilis screening as a part of HIV monitoring among HIV-positive MSM will have important epidemiological significance for the management of infected patients, and can help reduce the spread of syphilis.

## Introduction

Human immunodeficiency virus (HIV) infection among men who have sex with men (MSM) was a significant public health challenge worldwide (1). HIV continued to disproportionately affect MSM living in China, despite the introduction of highly active antiretroviral therapies (2). By the end of 2019, there had been 962,809 people living with HIV (PLWH) in China, and male-to-male sexual contact had become one of the main routes of HIV transmission (23.3%) (3). In addition to HIV, MSM, including HIV-positive ones, were disproportionally affected by sexually transmitted diseases (STD), especially syphilis (4).

Previous studies have shown that the prevalence of syphilis has risen among HIV-positive MSM (2, 5, 6). Syphilis and HIV co-infection can pose considerable health burdens to HIV-positive MSM because syphilis can elevate HIV viral load and decreases CD4 count in HIV-positive patients, thus increasing the risk of HIV-transmission to their serodiscordant partners (7). The increasing number of syphilis cases highlights the importance of syphilis control in HIV-positive MSM, not just for the health of the HIV patient, but also for the prevention of further transmission of HIV and syphilis among uninfected MSM (8). Early detection and treatment of syphilis and other curable STD had become a major component of comprehensive HIV care programs (9). The US Centers for Disease Control (CDC) and Prevention recommends at least annual screening for syphilis for HIV-positive MSM (10). However, most regions in China did not provide syphilis screening services for HIV-positive people, and the testing rate of syphilis was only 37.1% in China (11). Therefore, there is an urgent need to improve routine syphilis screening in HIV-positive MSM in China, which is essential in understanding the driving force of syphilis among HIV-positive MSM.

There is an unique environment to conduct this study in Guangdong Province of China. First, as one of the most developed provinces in China, Guangdong province is one of the few provinces in China that provides regular syphilis screening for HIV-positive MSM. Also, Guangdong has the largest number of HIV-positive MSM in China (12). Second, Guangdong Province has an increasingly rapid prevalence of syphilis where the prevalence of syphilis is 24.7% among MSM (13). However, few studies have been conducted to determine the prevalence and risk factors of syphilis among HIV-positive MSM in Guangdong, previous studies only focused on one city or with small simple sizes. This study aims to evaluate the prevalence of syphilis and determine the risk factors of syphilis among HIV-positive MSM in Guangdong Province.

## Methods

### Study design and participants

This cross-sectional study was conducted from June 2020 to August 2021 using both online and offline surveys. The online survey was carried out through WenJuanXing (Changsha Ranxing Information Technology, China), an electronic survey platform that was widely used and provided anonymous surveys in China. The offline survey was performed at HIV treatment clinics. The online and offline surveys were both conducted in six cities (Guangzhou, Shenzhen, Zhuhai, Dongguan, Foshan, and Zhongshan) in Guangdong Province. Guangdong Province has been consistently ranked first in the nation for syphilis and other STDs incidence estimation over the last decade. These cities were chosen based on the high prevalence of STD and local sexually transmitted infection (STI)/HIV prevention capabilities.

All potential participants were screened for eligibility after signing an electronic informed consent. Chinese men were eligible to participate if they were (1) born as male, (2) aged over 18 years old, (3) ever engaged in anal or oral sex with a man, (4) newly diagnosed as HIV positive in the last 2 years (since May 2018) and (5) ever tested for syphilis after being diagnosed with HIV. Those participants who did not sign informed consent will be excluded.

### Sample size

The primary outcome of our study was the prevalence of syphilis among HIV-positive MSM who had been tested for syphilis. A previous study reported that the prevalence of syphilis was 11.3% in China (2). We applied the two-sided confidence interval (CI) for One Proportion method to estimate a sample size of 655 for this study to produce a two-sided 95% CI and a width of 0.050. Therefore, at least 655 MSM need to be finally included in this study.

### Data collection

Electronic questionnaires were used for data collection in this study. The questionnaire items in this study were created based on discussions with HIV and STI experts, HIV-positive MSM, and policy makers in China. We also piloted the survey with 20 volunteer HIV-positive MSM to test questionnaire items. The pilot data were not included in the final analysis.

The questionnaire data were collected using both online and offline surveys in this study. For the online survey, it was conducted by trained staffs from the CDC in each city. The staffs sent the survey link to potential participants through mobile phone text messages or WeChat (the most popular messaging app in China). The offline surveys were conducted by trained doctors at designated HIV treatment clinics in each city. Each potential participant was voluntarily subjected to an electronic questionnaire survey. All questionnaires were filled out by HIV-positive MSM themselves. The offline survey was also performed through the same questionnaire link as the online survey, which can avoid duplication of data. To minimize the risk of duplicate participation from the same person, we allowed each survey link to be accessed only once by a single IP address, phone number and WeChat account.

### Measurements

#### Sociodemographic and behavioral variables

The collected information for social-demographic characteristics included gender, age, legal marital status, highest educational attainment, annual income (US dollar), and sexual orientation. Sexual behaviors included the number of male sexual partners and whether used condom frequently with male sex partners in the last 6 months. The main location to meet with the male sexual partners in the last 6 months was also recorded.

#### STD variables

STD variables included syphilis, chlamydia, gonorrhea, herpes, and human papillomavirus (HPV) statuses after HIV diagnosis. STD in this study was defined as the diagnosis of any of the following diseases in the past 2 years, syphilis, chlamydia, gonorrhea, herpes and HPV infection. These data were collected based on self-report.

#### Other variables

We also collected other variables including whether have had underlying diseases (diabetes, hypertension, tumor, pulmonary diseases, cardiac diseases, and kidney diseases) caused by HIV infection and the influence of Corona Virus Disease 2019 (COVID-19) on the HIV treatment.

### Statistical analysis

Descriptive analysis was conducted to describe the demographic characteristics, sexual behaviors and STD. A series of univariate and multivariate logistic regressions were conducted to explore the factors associated with syphilis and STD, respectively. In the multivariate model, we adjusted for age, legal marital status, highest educational attainment, annual income and sexual orientation. These adjusted variables were chosen according to our previous experience. All analyses were conducted on SAS (Version 9.4, SAS Institute Inc., Cary, NC).

## Results

### Study participants characteristics

Overall, a total of 944 participants were recruited in this study. About half of the participants were between 26 and 35 years old (44.1%, 416/944) and had a College/Bachelor's or above degree (54.6%, 515/944). The majority of participants were never married (81.9%, 773/944) and gay, bisexual, and other MSM (GBMSM) (94.1%, 888/944). Around one-third of participants had an annual income between US dollar 5,001 and US dollar 10,000 (37.1%, 350/944) (Table 1).

**Table 1:** Social-demographic and sexual behavioral characteristics among HIV-positive MSM in Guangdong Province from June 2020 to August 2021 (*N* = 944).

**Variables**	**Total** **(*N* = 944, %)**	**Positive** **(*N* = 141, %)**	**Negative** **(*N* = 803, %)**	**χ^2^**	***P***
**Demographic characteristics**					
**Age**					
18–25	357 (37.8)	43 (30.5)	314 (39.1)	3.80	0.150
26–35	416 (44.1)	70 (49.6)	346 (43.1)		
≥36	171 (18.1)	28 (19.9)	143 (17.8)		
**Legal marital status**					
Never married	773 (81.9)	114 (80.8)	659 (82.1)	7.34	**0.025**
Engaged / Married	84 (8.9)	7 (5.0)	77 (9.6)		
Separated / Divorced /Widowed	87 (9.2)	20 (14.2)	67 (8.3)		
**Highest educational attainment**					
Middle school or below	178 (18.8)	36 (25.5)	142 (17.7)	10.063	**0.007**
High school	251 (26.6)	45 (31.9)	206 (25.6)		
College/Bachelors or above	515 (54.6)	60 (42.6)	455 (56.7)		
**Annual income (US dollar)**					
< 5,000	152 (16.1)	33 (23.4)	119 (14.8)	9.252	**0.026**
5,001–10,000	350 (37.1)	54 (38.3)	296 (36.8)		
10,001–15,000	246 (26.1)	34 (24.1)	212 (26.4)		
>15,000	196 (20.7)	20 (14.2)	176 (22.0)		
**Sexual orientation**					
GBMSM	888 (94.1)	134 (95.1)	754 (93.9)	0.278	0.598
Cisgender	56 (5.9)	7 (4.9)	49 (6.1)		
**Where did you mainly meet with your male sexual partners in the last 6 months**					
**Pub, disco, tearoom, or club**					
Yes	37 (3.9)	2 (1.4)	35 (4.4)	2.75	0.097
No	907 (96.1)	139 (98.6)	768 (95.6)		
**Spa or bath house, sauna, foot or body massage parlor**					
Yes	21 (2.2)	7 (5.0)	14 (1.7)	4.34	**0.037**
No	923 (97.8)	134 (95)	789 (98.3)		
**Park, public restroom, public lawn**					
Yes	13 (1.4)	4 (2.8)	9 (1.1)	1.49	0.222
No	931 (98.6)	137 (97.2)	794 (98.9)		
**Website/social media**					
Yes	537 (56.9)	73 (51.8)	464 (57.8)	1.77	0.184
No	407 (43.1)	68 (48.2)	339 (42.2)		
**Through friends**					
Yes	80 (8.5)	7 (5.0)	73 (9.1)	2.63	0.105
No	864 (91.5)	134 (95.0)	730 (90.9)		
**Sexual behavior**					
**Number of male sexual partners in the last 6 months** ^**a**^					
1	220 (46.3)	26 (40.0)	194 (47.3)	1.21	0.272
>1	255 (53.7)	39 (60.0)	216 (52.7)		
**Frequently condom use with male sex partners in the last 6 months**					
0% condom use	17 (3.6)	4 (6.1)	13 (3.2)	1.60	0.661
< 50% condom use	62 (13.0)	9 (13.9)	53 (12.9)		
More than 50% condom use	101 (21.3)	14 (21.5)	87 (21.2)		
100% condom use	295 (62.1)	38 (58.5)	257 (62.7)		
**Diseases**					
**Are your underlying diseases caused by the HIV infection** ^**b**^					
Yes, some of them	10 (8.8)	4 (23.5)	6 (6.3)	8.94	**0.030**
Yes, almost all of them	13 (11.5)	0 (0.0)	13 (13.5)		
No	50 (44.3)	5 (29.4)	45 (46.9)		
Not sure/Do not know	40 (35.4)	8 (47.1)	32 (33.3)		
**Did the influence of COVID-19 on your HIV treatment get resolved** ^**c**^					
Yes	490 (84.6)	69 (78.4)	421 (85.7)	3.09	0.079
No	89 (15.4)	19 (21.6)	70 (14.3)		

^a^This analysis was restricted to participants who had sexual partners in the 6 months.

^b^This analysis was restricted to participants who had underlying diseases.

^c^This analysis was restricted to participants whose HIV treatment influence by COVID-19. GBMSM, Gay, bisexual, and other MSM.

The bold values indicates the *P* values < 0.05.

### Sexual behaviors

About half of the participants had more than one sexual partner among 475 participants who had sexual partners in the last 6 months (53.7%, 255/475). The majority of the participants always used condom with male sex partners in the last 6 months (62.1%, 295/475). More than half of the participants sought sexual partners through website or social media (56.9%, 537/944) in the last 6 months (Table 1).

### Prevalence of STD

In this study, we found that 14.9% of the participants were ever diagnosed with syphilis, 8.5% ever diagnosed with HPV, 1.4% ever diagnosed with herpes, 0.9% ever diagnosed with gonorrhea, 0.2% ever diagnosed with chlamydia and 23.3% ever diagnosed with STD in the past 2 years. In addition, 1.8% of the participants were ever diagnosed with two STDs and only three participants had more than two STDs in the last 2 years (Table 2).

**Table 2:** Prevalence of STD among HIV-positive MSM in Guangdong Province from June 2020 to August 2021 (*N* = 944).

**Diseases**	***N* (%)**	**95% CI**
Syphilis	141 (14.9)	12.7–17.2
HPV	80 (8.5)	6.7–10.3
Herpes	13 (1.4)	0.6–2.1
Gonorrhea	8 (0.9)	0.3–1.4
Chlamydia	2 (0.2)	0.0–0.5
STD*	220 (23.3)	20.6–26.0
Co-infection with two diseases	17 (1.8)	1.0–2.6
Co-infection with three diseases	2 (0.2)	0.0–0.5
Co-infection with four diseases	1 (0.1)	0.0–0.3

Any of syphilis, chlamydia, gonorrhea, herpes, and HPV.

The ^*^ symbol indicates any of syphilis, chlamydia, gonorrhea, herpes, and HPV.

### Factors associated with syphilis

After adjusted for age, marital status, education, annual income and sexual orientation, the multivariate logistic regression analysis indicated that the participants who met male sexual partners mainly through spa or bath house, sauna, foot or body massage parlor in the last 6 months [adjusted Odds Ratio (*aOR*) = 2.91, 95% CI: 1.09–7.79], who were diagnosed with herps after HIV diagnosis (a*OR* = 3.79, 95% CI: 1.16–12.39), and whose influence of COVID-19 on the HIV treatment was resolved (a*OR* = 1.91, 95% CI: 1.04–3.49) were more likely to be diagnosed with syphilis. The participants who had not underlying diseases caused by the HIV infection (a*OR* = 0.11, 95% CI: 0.01–0.92) were less likely to have syphilis (Table 3).

**Table 3:** Factors associated with syphilis among HIV-positive MSM in Guangdong Province from June 2020 to August 2021 (*N* = 944).

**Variables**	**cOR (95% CI)**	***P***	**aOR (95% CI)^#^**	***P***
**Where did you mainly meet with your male sexual partners in the last 6 months**				
**Pub, disco, tearoom, or club**				
Yes	0.32 (0.08–1.33)	0.116	0.31 (0.07–1.34)	0.116
No	*Ref*		*Ref*	
**Spa or bath house, sauna, foot or body massage parlor**				
Yes	2.94 (1.17–7.43)	0.022	2.91 (1.09–7.79)	**0.033**
No	*Ref*		*Ref*	
**Park, public restroom, public lawn**				
Yes	2.58 (0.78–8.48)	0.120	2.07 (0.57–7.47)	0.269
No	*Ref*		*Ref*	
**Website/social media**				
Yes	0.78 (0.55–1.12)	0.185	0.80 (0.55–1.15)	0.227
No	*Ref*		*Ref*	
**Through friends**				
Yes	0.52 (0.24–1.16)	0.110	0.56 (0.25–1.25)	0.158
No	*Ref*		*Ref*	
**Number of male sexual partners in the last 6 months**				
1	*Ref*		*Ref*	
>1	1.35 (0.79–2.3)	0.273	1.36 (0.78–2.36)	0.281
**Frequently condom use with male sex partners in the last 6 months**				
0% condom use	*Ref*		*Ref*	
< 50% condom use	0.55 (0.15–2.08)	0.379	0.45 (0.11–1.81)	0.260
More than 50% condom use	0.52 (0.15–1.83)	0.311	0.42 (0.11–1.59)	0.201
100% condom use	0.48 (0.15–1.55)	0.220	0.40 (0.12–1.38)	0.147
**Diagnosed with gonorrhea after the HIV diagnosis**				
Yes	3.47 (0.82–14.68)	0.091	3.2 (0.69–14.8)	0.138
No	*Ref*		*Ref*	
**Diagnosed with herpes after the HIV diagnosis**				
Yes	3.65 (1.18–11.33)	**0.025**	3.79 (1.16–12.39)	**0.028**
No	*Ref*		*Ref*	
**Diagnosed with HPV after the HIV diagnosis**				
Yes	1.12 (0.6–2.08)	0.731	1.07 (0.57–2.03)	0.835
No	*Ref*		*Ref*	
**Do you have any other underlying diseases**				
Yes	1.03 (0.6–1.79)	0.904	1.05 (0.59–1.86)	0.868
No	*Ref*		*Ref*	
**Are your underlying diseases caused by the HIV infection**				
Yes	*Ref*		*Ref*	
No	0.17 (0.04–0.8)	**0.025**	0.11 (0.01–0.92)	**0.042**
Not sure/Do not know	0.38 (0.09–1.65)	0.195	0.26 (0.03–2.03)	0.200
**Did the influence of COVID-19 on your HIV treatment get resolved**				
Yes	*Ref*		*Ref*	
No	1.66 (0.94–2.92)	0.081	1.91 (1.04–3.49)	**0.036**

^#^All variables were adjusted for age, legal marital status, highest educational attainment, monthly income and sexual orientation. cOR, crude Odds Ratio; aOR, adjusted Odds Ratio.

The bold values indicates the *P* values < 0.05.

### Factors associated with STD

After adjusted for age, marital status, education, annual income and sexual orientation, the multivariate logistic regression analysis indicated that the participants who met male sexual partners mainly through spa or bath house, sauna, foot or body massage parlor in the last 6 months (a*OR* = 2.55, 95% CI: 1.01–6.42), mainly through park, public restroom, public lawn in the last 6 months (a*OR* = 3.87, 95% CI: 1.19–12.57), and had more than one male sexual partner in the last 6 months (a*OR* = 1.88, 95% CI: 1.17–3.02) were more likely to have STD. The participants who had no underlying diseases caused by the HIV infection (a*OR* = 0.17, 95% CI: 0.03–0.90) were less likely to have STD (Table 4).

**Table 4:** Factors associated with STD among HIV-positive MSM in Guangdong Province from June 2020 to August 2021 (*N* = 944).

**Variables**	**cOR (95% CI)**	***P***	**aOR (95% CI)**	***P***
**Where did you mainly meet with your male sexual partners in the last 6 months**				
**Pub, disco, tearoom, or club**				
Yes	0.76 (0.33–1.76)	0.521	0.71 (0.3–1.7)	0.443
No	*Ref*		*Ref*	
**Spa or bath house, sauna, foot or body massage parlor**				
Yes	2.53 (1.05–6.09)	**0.038**	2.55 (1.01–6.42)	**0.047**
No	*Ref*		*Ref*	
**Park, public restroom, public lawn**				
Yes	3.93 (1.31–11.83)	**0.015**	3.87 (1.19–12.57)	**0.024**
No	*Ref*		*Ref*	
**Website/social media**				
Yes	0.82 (0.61–1.11)	0.206	0.83 (0.61–1.14)	0.247
No	*Ref*		*Ref*	
**Through friends**				
Yes	0.62 (0.33–1.14)	0.122	0.66 (0.35–1.23)	0.187
No	*Ref*		*Ref*	
**Number of male sexual partners in the last 6 months**				
1	*Ref*		*Ref*	
>1	1.85 (1.17–2.92)	**0.009**	1.88 (1.17–3.02)	**0.009**
**Frequently condom use with male sex partners in the last 6 months**				
0% condom use,	*Ref*		*Ref*	
< 50% condom use	0.86 (0.24–3.09)	0.820	0.83 (0.22–3.13)	0.781
More than 50% condom use	1.19 (0.36–3.95)	0.781	1.13 (0.32–4.00)	0.848
100% condom use	0.78 (0.25–2.48)	0.671	0.76 (0.23–2.55)	0.656
**Do you have any other underlying diseases**				
Yes	*Ref*		*Ref*	
No	0.72 (0.46–1.12)	0.144	0.68 (0.43–1.07)	0.097
**Are your underlying diseases caused by the HIV infection**				
Yes	*Ref*		*Ref*	
No	0.17 (0.04–0.71)	**0.015**	0.17 (0.03–0.90)	**0.037**
Not sure/Don't know	0.32 (0.08–1.34)	0.119	0.41 (0.08–2.07)	0.280
**Did the influence of COVID-19 on your HIV treatment get resolved**				
Yes	*Ref*		*Ref*	
No	1.05 (0.63–1.76)	0.850	1.12 (0.65–1.92)	0.685

^#^All variables were adjusted for age, legal marital status, highest educational attainment, monthly income and sexual orientation. cOR, crude Odds Ratio; aOR, adjusted Odds Ratio.

The bold values indicates the *P* values < 0.05.

## Discussion

Syphilis is a growing public health concern and the prevalence of syphilis is increasing among HIV-positive MSM in all countries over the world (14). This study expands the literature by focusing on a large number of HIV-positive MSM in China, exploring the prevalence and factors of syphilis, and describing the prevalence of other STDs. Our study suggests that the prevalence of syphilis and other STDs was relatively high, and seeking male sexual partners through traditional meeting places and reported a higher number of sexual partners were positively associated with syphilis and STD diagnoses among HIV-positive MSM in China.

We found that about one-seventh of HIV-positive MSM in Guangdong province had syphilis in the last 2 years. This prevalence of syphilis is higher than that reported in a previous study among HIV-positive MSM in China (2, 5), but lower than that previously reported in Turkey (4). There are several reasons for the high syphilis prevalence among HIV-positive MSM in our study. First, only 62.1% of participants used condoms consistently in this study, and the high prevalence was attributed to unprotected anal intercourse. Second, HIV impairs the immune system in ways that make it easier for syphilis to take hold (15). Third, poor awareness of syphilis prevention is also an important reason leading to high syphilis incidence among HIV-positive MSM (16). The high syphilis prevalence alongside highly risky sexual behaviors among HIV-positive MSM highlights the importance of syphilis control in China. A previous study has unveiled that frequent testing is a cost-effective way to reduce the potential onward transmission of syphilis (17). Hence, including routine syphilis testing as part of HIV monitoring in HIV-positive MSM can help reduce the duration of infectiousness and incidence of syphilis. Additionally, syphilis self-testing can help improve testing uptake among key populations and self-testing kits are also very easy to obtain online in China (18). It is also very necessary to promote syphilis self-testing among HIV-positive MSM in China.

We also observed that about one-fifth of the HIV-positive MSM have been diagnosed at least one of the listed STDs in the last 2 years in this study. This prevalence of STD is much higher than that reported in a previous study conducted among generally HIV-infected adults in China (19), and the results from a global meta-analysis among PLWH (20). Given the high prevalence of STD and the fact that many STI are asymptomatic infection, our findings highlight the necessity of carrying out regular STD testing among HIV-positive MSM, but the previous study demonstrated that the STD testing rate among HIV-positive MSM still remained low (10). To increase the STD testing, the US CDC launched a guideline to recommend MSM, especially HIV-positive MSM take at least one STD testing annually (21), yet such a guideline is not available in China. Hence, there is an urgent need to introduce national policies to demand MSM patients to be tested STD at least once per year, and to increase the frequency of testing depending on the patient's sexual activities. In addition, it is also necessary to ensure that medical providers can provide regular STD testing follow-up and consulting services for HIV-infected patients.

It was found that MSM who mainly seek male sexual partners through traditional meeting places such as spa or bathhouse, sauna, foot or body massage parlor were more likely to have syphilis. This is consistent with other studies in China (22). The previous study has evidenced that MSM among different venues (i.e., parks, public baths, bars/clubs, or internet) had different high-risk sexual behaviors and HIV/STI risks (23). Participants who seek sexual partners through traditional meeting places have more sexual partners and unprotected sexual behaviors. Comprehensive interventions including condoms and lubrication distribution, health education and providing counselors and special events related to safer-sex skills building need to be taken in traditional meeting places (24). This study also found that men with higher number of sexual partners have a higher risk of STI, which is consistent with other studies (25). Hence, this finding highlights the importance of enhancing sexual health services among HIV-positive MSM, especially those with a high prevalence of STD. In addition, we also found MSM whose influence of COVID-19 on the HIV treatment was resolved were more likely to be diagnosed with syphilis. Travel restrictions during the COVID-19 epidemic in China have impacted on the antiretroviral therapy of HIV-positive MSM (26). The participants whose influence of COVID-19 on the HIV treatment was resolved may have more high-risk behaviors. In order to reduce the negative impact on HIV-positive MSM, more attention should be paid to conducting health behavior education during the COVID-19 epidemic.

This study has several limitations. First, all the data were collected through self-report, the syphilis and other STDs prevalence were not determined by serum testing, which may result in information bias. Second, this study was a cross-sectional study. The cross-sectional nature of the design limits causal inference. Third, since HIV-positive MSM are a hard-to-reach population, we were not able to sample them randomly. However, our study has covered most HIV-positive MSM in Guangdong Province and the sample was representative. Fourth, this study recruited HIV-diagnosed MSM newly in the past 2 years, the results may not be able to generalize to other HIV-positive MSM over 2 years period.

## Conclusion

The prevalence of syphilis and other STDs is relatively high among HIV-positive MSM in China. The finding of this study highlights the need to establish a national guideline for STD screening among HIV-positive MSM in China. Routine syphilis screening as part of HIV monitoring in HIV-positive MSM will have important epidemiological significance for the management of infected patients, and can help reduce the duration of infectiousness and incidence of syphilis.

## Data availability statement

The raw data supporting the conclusions of this article will be made available by the authors, without undue reservation.

## Ethics statement

The studies involving human participants were reviewed and approved by the Ethical Committee of Guangzhou Center for Disease Control and Prevention (GZCDC-ER-P2019001). The patients/participants provided their written informed consent to participate in this study.

## Author contributions

PZ, JZ, and WT wrote and reviewed the manuscript. WT and XF collected the data. ZY and JC helped analyze data. ZY and YZ helped collect the data and reviewed the manuscript. All authors contributed to the article and approved the submitted version.

## Funding

This work was supported by the National Natural Science Foundation of China (Grant Numbers: 82173619 and 81773544), the Science and Technology Planning Project of Guangdong Province (Grant Number: 2020B1212030008), and Medical Scientific Research Foundation of Guangdong Province (Grant Numbers: B2021297 and B2022139).

## Conflict of interest

The authors declare that the research was conducted in the absence of any commercial or financial relationships that could be construed as a potential conflict of interest.

## Publisher's note

All claims expressed in this article are solely those of the authors and do not necessarily represent those of their affiliated organizations, or those of the publisher, the editors and the reviewers. Any product that may be evaluated in this article, or claim that may be made by its manufacturer, is not guaranteed or endorsed by the publisher.
